# Defining Amaranth, Buckwheat and Quinoa Flour Levels in Gluten-Free Bread: A Simultaneous Improvement on Physical Properties, Acceptability and Nutrient Composition through Mixture Design

**DOI:** 10.3390/foods11060848

**Published:** 2022-03-16

**Authors:** Etiene Valéria Aguiar, Fernanda Garcia Santos, Ana Carolina Ladeia Solera Centeno, Vanessa Dias Capriles

**Affiliations:** Laboratory of Food Technology and Nutrition, Department of Biosciences, Institute of Health and Society, Campus Baixada Santista, Federal University of São Paulo (UNIFESP), Santos 11015-020, Brazil; etiene.aguiar@unifesp.br (E.V.A.); fg.santos@unifesp.br (F.G.S.); anacladeia@gmail.com (A.C.L.S.C.)

**Keywords:** gluten-free, pseudocereals, whole flour, bread quality, response surface methodology, multiple factor analysis

## Abstract

The study aimed to define the ideal proportions of pseudocereal flours (PF) in sensory-accepted gluten-free bread (GFB) formulations. The characteristics of GFB developed with PF (amaranth, buckwheat, and quinoa) were verified through a mixture design and response surface methodology. Three simplex-centroid designs were studied to analyze the effects of each PF and their interactions with potato starch (PS), and rice flour (RF) on GFB’s physical and sensory characteristics, each design producing three single, three binary and six ternary GFB formulations. Results showed that using PF alone resulted in unacceptable GFB. However, the interactions between PF and RF improved the loaf specific volume and the crumb softness and also enhanced appearance, color, odor, texture, flavor, and overall liking. Moreover, the composite formulations prepared with 50% PF and 50% RF (flour basis) presented physical properties and acceptability scores like those of white GFB, prepared with 100% RF or a 50% RF + 50% PS blend (flour basis). Maximum proportions of PF to obtain well-accepted GFB (scores ≥7 for all evaluated attributes on a 10-cm hybrid hedonic scale) were defined at 60% for amaranth flour (AF), 85% for buckwheat flour (BF), and 82% for quinoa flour (QF) in blends with RF.

## 1. Introduction

There is constantly growing demand for gluten-free (GF) products, projected to achieve an approximate global market of USD 24 billion by 2027 [1]. Despite the increase in GF food available on the market, individuals with restrictions on gluten consumption still report difficult access to these products, since they usually have high shelf prices, restricted variety and availability, and poor palatability [2,3].

Among all GF products, bread has been the most investigated by researchers in several countries and it is also the most requested by consumers with celiac disease [4]. However, gluten-free bread (GFB) is still considered to be a product with unsatisfactory texture and flavor, lacking in nutritional content and having a short shelf life [3,5].

GFB often presented poor nutritional composition because it is mostly made using refined raw materials like white rice flour blended with corn, potato and/or cassava starches. Although these raw materials are readily available, made with cheap ingredients, and have neutral color, flavor, and odor, they lack dietary fiber, vitamins, and minerals. They have high levels of available carbohydrates, resulting in products with high glycemic response and poor nutritional quality, since they are neither enriched nor fortified [4,5]. Thus, improvement in the nutritional composition of GFB is an important objective for food research and development, a challenge that is concomitant with the improvement of the technological and sensory characteristics of these products [6].

To improve GFB formulation, the use of alternative ingredients with a rich nutrient and bioactive compounds content, such as wholemeal pseudocereal flours obtained from naturally GF grains, has been recommended [7].

The pseudocereals amaranth, buckwheat, and quinoa present high protein content, notably essential amino acids, mainly lysine (limited in cereals) and sulphur amino acids (limited in legumes). In addition, the considerable fiber, vitamin, and mineral content, and their potential as functional food are factors that increase the interest in the use of these grains for human consumption [8]. Thus, the incorporation of pseudocereals in the formulation has the potential to enhance the nutritional profile of GFB, which can benefit the health of individuals with diseases related to gluten consumption [8].

Several studies have been conducted showing that it is possible to use pseudocereal flours (PF) in GFB formulations [9,10,11,12,13,14]. However, to date, no publications report the effects of different pseudo cereal flour levels on physical characteristics, degree of liking, and nutritional profile of GFB. Therefore, this study aimed to evaluate the maximum limits and the ideal proportions of PF (amaranth (AF), buckwheat (BF) and quinoa (QF)) in combination with rice flour (RF) and potato starch (PS) using a mixture design to obtain GFB with improved technological, sensory, and nutritional properties.

## 2. Materials and Methods

### 2.1. Ingredients

The grains of amaranth (*Amaranthus caudatus*) and quinoa (*Chenopodium quinoa*), originally from Peru, were obtained from RS Blumos Industrial e Comercial Ltd.—Cotia-SP, Brazil, while the grains of buckwheat (*Fagopyrum esculentum*), originally from Bolivia, were obtained from Estação dos Grãos Ltd.—São Paulo, Brazil. The pseudocereal grains were transported to the Food Technology and Nutrition Laboratory (LAbTAN, UNIFESP) and milled using a mill (Laboratory Mill 3303, Perten Instruments, Stockholm, Sweden) at level 0, obtaining flours with the smallest particle size possible, in order to not confer or minimize the sensation of grit, which is often mentioned by consumers when tasting GFB developed with wholemeal flours [15]. Among the PF, the AF presents the largest particle size (83% ≥ 250 μm), followed by the QF (73% ≥ 250 μm), and the BF with the smallest particle size (60% < 180 μm) [16].

The xanthan gum (Ziboxan F80, Deosen Biochemical Ltd.—Mongolia, China) was donated by the company Vogler Ingredients Ltd. (São Bernardo do Campo-SP, Brazil) while carboxymethylcellulose (Denvercel FG-2504A, Denver Especialidades Químicas Ltd.—Cotia-SP, Brazil) was donated by its manufacturer. Other ingredients used for GFB preparation were obtained at the local market.

### 2.2. Methods

#### 2.2.1. Formulation and Production of Gluten-Free Breads

The GFB formulation were elaborated according to Aguiar et al. (2021b) [16] and consisted of the following ingredients on a flour basis (f.b.): 100% blend of one PF with RF and/or PS, according to a mixture design, 25% egg, 10.5% whole milk powder, 6% sugar, 6% soybean oil, 2% salt, 0.8% dry yeast, 0.3% xanthan gum, 0.3% carboxymethylcellulose and 100% water.

The straight dough method was used as reported by Aguiar et al. (2021b) [16]. The analyses were conducted within up to 3 h after production. Twelve loaves of each GFB experimental formulation were produced, in two batches. Six loaves were used for the analysis of physical properties and the other six were used in the sensory analysis.

#### 2.2.2. Experimental Design

Three simplex centroid experimental designs were conducted, combining each PF with the RF and PS: different percentage mixes of (A) AF with RF + PS; (B) BF with RF + PS; (C) QF with RF + PS. For each of the three designs, there were twelve experimental formulations, three constituted of single components (100%), three of binary blends prepared with 50% of each of two components, one formulation of ternary blend consisting of the combination of 33.3% of each component, representing the central point of the model, which was made in three repetitions, and three formulations corresponding to the ternary blend consisting of the combination of 66% of one component and 17% of each of the others, corresponding to the axial points (Figure S1, on Supplementary Material). The sequence of execution of the experiments was randomized by a prior draw. The highest content level of each component in the blend of flours and starches (proportion = 1) represented 35.8 g of the dough (Figure S1, on Supplementary Material).

#### 2.2.3. Bread Quality Evaluation

Physical properties were analyzed as described by Aguiar et al. (2021b) [16]. The analyses of specific loaf volume, crumb firmness and moisture content were made, respectively, according to method 10-05.01, 74-09 and 44-15.02 of AACC (2010) [17], while the crumb cell structure was analyzed according to Santos et al. (2020) [18].

The sensory acceptance of samples was conducted in ten sensory analysis sessions randomized for each design, offering in each session up to three samples of the same design, with balanced order of presentation.

In each sensory analysis session, 50 bread consumers, recruited from students and staff from the university campus, aged 18–59 years, assessed the acceptability of the attributes: appearance, color, odor, texture, flavor and overall liking of the breads, on a semi-structured 10 cm hybrid hedonic scale (0 = disliked very much, 5 = neither liked/nor disliked, 10 = liked very much) [19].

The evaluators received the samples of bread (slices of 12 mm in thickness) monadically, packaged in polypropylene bags and coded with three random digits. The participants assessed the GFB formulations in individual booths in the Sensory Analysis Laboratory, being instructed to drink water between samples to minimize residual effects.

#### 2.2.4. Selection of Samples and Quality Verification

The GFB physical properties and acceptability served as response variables for the mixture design regression models, applying the Scheffé canonical polynomial models as explained by Aguiar et al. (2021b) [16].

Principal component analysis (PCA) also contributed to determining the content levels of these flours that do not alter the physical properties and acceptability compared with the control GFB formulation.

#### 2.2.5. Characterization of Selected Samples

The selected samples, containing both optimum and maximum levels of PF, had their dough thermomechanical characteristics evaluated using the Chopin + 90 protocol in Mixolab^®^ (Chopin Technologies, Villeneuve-la-Garenne, France), in which all ingredients (except yeast) were mixed in the proportions utilized in the bread preparation, using a total of 90 g of dough. Adaptations were made to method 54-60.01 of AACC (2010) [17] to allow knowing the effect of ingredients on the dough characteristics, subject to mixing and temperature variation, simulating the breadmaking process. The same parameters as those reported by of Santos et al. (2021) [20] were observed here: initial consistency (C1), weakening of protein network (C2), maximum (C3) and minimum (C4) peak during the heating phase and the value obtained after cooling (C5). Two repetitions were performed for each sample.

In addition, the selected formulations were prepared and analyzed experimentally to verify the physical properties and sensory acceptance using, respectively, the methods cited on Section 2.2.3, these results being compared statistically with the expected values of the fitted models.

The selected GFB formulations had the proximate composition analyzed. Moisture, ash, protein and lipid contents were analyzed following the respective methods 950.46, 923.03, 960.52, 920.39 of AOAC (2005) [21]. Dietary fiber (soluble and insoluble) content was verified utilizing the enzymatic-gravimetric method 991.43 of AOAC (2005) [21] and analytical kit K-ACHDF (Megazyme International Ireland Ltd., Bray, Ireland). Available carbohydrates were calculated by difference. Data were means of three repetitions and expressed as g/100 g of GFB.

#### 2.2.6. Ethical Considerations

This study was approved by the Research Ethics Committee of UNIFESP (protocol number 1.814.143) and all the participants signed an informed consent form before enrollment in the research.

#### 2.2.7. Statistical Analysis

The adequacy of the mixture regression model was verified through variance analysis (*F* test), *R*^2^ values, lack-of-fit test, and diagnostic plots such as normal and residual plots. One-way analysis of variance (ANOVA) and Tukey’s test were used to verify the differences in treatment means, comparing physical properties, sensory acceptance scores and centesimal composition of the selected GFB formulations. The Statistica 12.0 statistical software (StatSoft Inc., Tulsa, OK, USA, 2013) was used for data processing.

Multiple factor analysis (MFA) was utilized to investigate the relationships of the studied variables (physical, sensory and Mixolab parameters) using the XLSTAT 2021.2 software (Addinsoft, New York, NY, USA), with the significance level established at 0.05 for all analyses.

## 3. Results

### 3.1. Mixture Design and Response Surface Analysis

**Figure 1:**
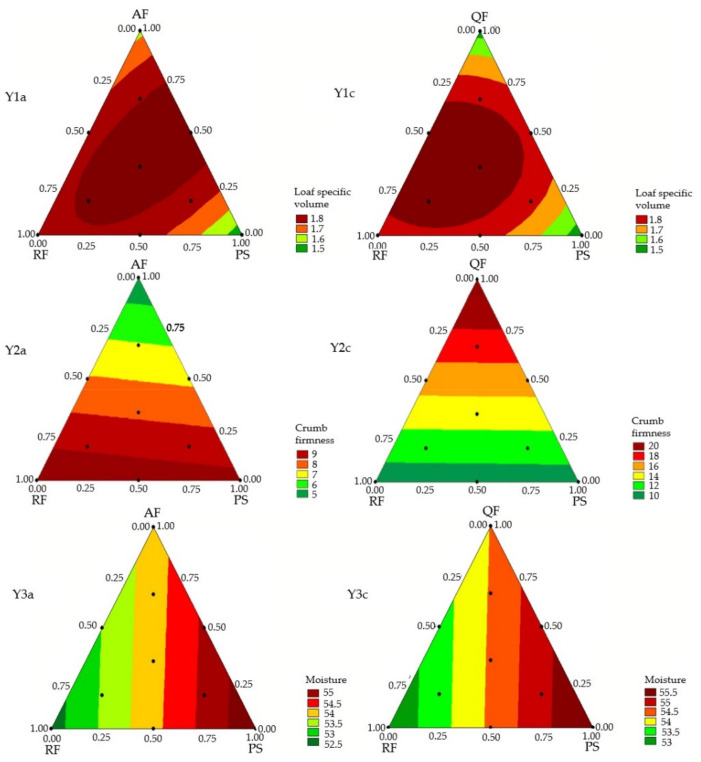
Contour plots from the predict model equations for physical properties of the gluten-free bread based on a mixture design. Formulation ID: AF—amaranth flour; BF—buckwheat flour; QF—quinoa flour; RF—rice flour; PS—potato starch.

**Figure 2:**
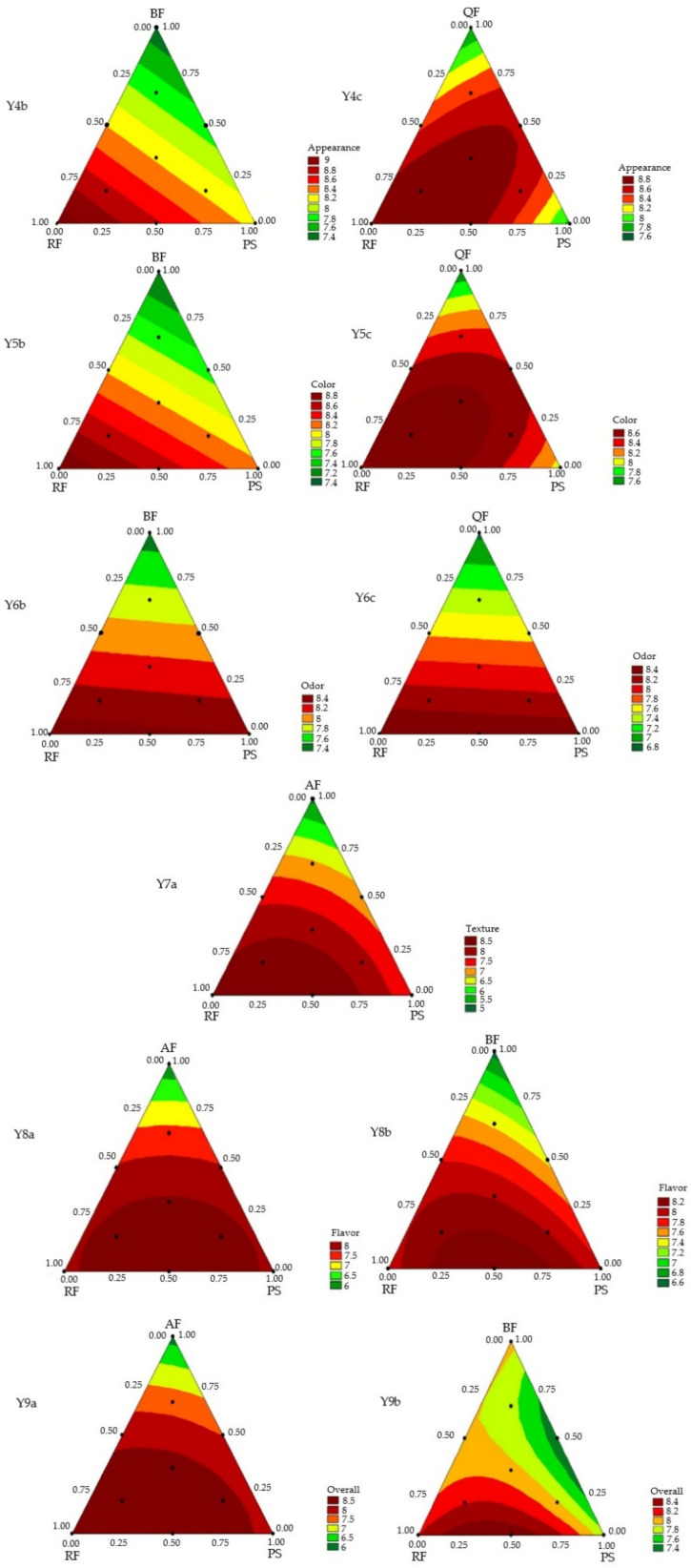
Contour plots from the predict model equations for the sensory acceptability scores (10-cm hybrid hedonic scale) of the gluten-free breads based on a mixture design. Formulation ID: AF—amaranth flour; BF—buckwheat flour; QF—quinoa flour; RF- rice flour; PS—potato starch.

Table 1 presents the mixture regression models obtained for the physical properties and for the acceptability of the GFB. The models obtained with designs A and C were significant for the physical properties. For the acceptability scores, only models obtained with designs B and C were significant for appearance, color, and odor; only design A presented a significant model for texture, while models from designs A and B were fitted for overall liking and flavor acceptance. Linear and quadratic models were obtained but no ternary interaction was significant for the variables studied. The significant models, without lack of fit and with high coefficient of determination (*R*^2^_adj_), with 70 to 98% of the experimental variability being explained by the models, were used to generate the contour curves (Figure 1 and Figure 2).

The loaf specific volume and crumb firmness are related to the sensory attributes of the bread [22,23] and were therefore evaluated more thoroughly in this study. The objective was to obtain bread with higher expansion and lower crumb firmness, indicating a softer loaf.

For specific volume, as displayed in Table 1 and Figure 1, RF showed the higher coefficient values in the regression models, therefore being responsible for higher specific volume of loaves, while PS (lower coefficient value) promotes lower specific volume. The GFB formulation prepared only with BF presented the greatest value for specific volume, followed by the formulations prepared with RF, AF, QF, and PS. The binary blends of AF with PS, similarly to QF with RF or PS showed a synergistic effect, increasing the specific volume of the bread.

No significant interactions were found for the other blends. Samples with the highest values for specific volume (~1.8 cm^3^/g) are indicated in the experimental region in dark red (Figure 1), comprising different blends of two or three components containing 30–75% AF combined with RF or PS (Figure 1 Y1a), and containing 10–65% QF (Figure 1 Y1c) combined with RF or PS. The results show that it is possible to use up to 75% AF and up to 80% QF combined with RF and obtain GFB in the region of highest specific volume.

In general, formulations containing equivalent amounts of AF and QF show close values for specific volume, while formulations containing BF showed higher values (Figure S2, on Supplementary Material). Alvarez-Jubete, Arendt & Gallagher (2009) [9] verified a difference in pasting properties, observing that, between the PFs, BF presented the highest peak viscosity, which was associated with GFB with improved specific volume when compared with GFB containing AF or QF. BF presents a higher amount of amylose in the starch composition (>45%), which may contribute to a higher dough viscosity in formulations prepared with BF, increasing their capacity to retain gases, resulting in breads with improved volume [9]. For crumb firmness, AF showed the lowest coefficient in relation to the other single components. However, it is worth mentioning that the formulation prepared with 100% AF showed to be inadequate, as it presented very gummy texture, preventing the appropriate formation of a bread, as reported previously by Alvarez-Jubete, Arendt & Gallagher (2009) [9] in other conditions of formulation and processing. GFB developed with 100% AF showed inadequate characteristics. This could be due to the high starch content of the flour (65–75%), which has direct influence on the higher viscosity of the dough due to the gelatinization of this starch, in addition to the high protein content of the flour. The proteins present in AF have the capacity to form gels and the high concentration of these gels can affect the capacity of adequate development of alveoli in the dough [24]. The combination of AF with RF or PS causes dilution of this gel, enabling adequate development of crumb and contributing to an increase in specific volume and a decrease in crumb firmness.

QF showed the highest coefficient values in the regression models, therefore being responsible for the highest crumb firmness. The GFB formulation prepared only with BF showed the highest value for crumb firmness, followed by the formulations prepared with QF, RF, and PS. However, the blends of BF or QF with RF and/or PS had no significant effect on crumb firmness.

The results found in this study are similar to those reported by Föste et al. (2014) [25], as they also associate the presence of QF in GFB with increased crumb firmness, resulting in breads with lower softness. These effects may be related to the high amount of fibers present in QF, due to decreased starch gelatinization caused by the competition for water between fibers and starch [26].

Concerning crumb moisture, PS showed the highest coefficient values in the regression models, being responsible for the highest crumb moisture, while RF (lowest coefficient value) promoted the lowest crumb moisture, and AF, BF, and QF promoted intermediate values. Little variation was seen in crumb moisture between the 36 experimental formulations (variation from 51.6 to 55.6%), but no significant interactions were observed between the components of these blends in the crumb moisture of the GFB.

The variations in the crumb moisture can be associated with the differences in the properties of each flour, as they vary in the content and in the composition of the starch and protein fractions, as well as with the higher water absorption capacity of the PF and of PS compared with RF [27,28].

In general, for the GFB developed with equal PF proportions, BF enabled better expansion of the breads, providing, at all content levels, the formation of a more homogeneous crumb, with the highest number of small alveoli and a more even distribution. The use of QF enabled the formation of more uniform crumb in relation to those obtained with AF. The use of high proportions (66% and 100%) of AF resulted in bread with compact structure and crumb with few alveoli.

The contour charts for the attributes of acceptability of the GFB (Figure 2) show the possibility of using different blends of BF or QF with both RF and PS to obtain GFB with good acceptability of appearance, color, and odor (scores > 7). The results also show the synergistic effect of the blend of AF with RF or PS, which enables GFB with accepted texture. Blends of AF or BF with RF and PS enable increased flavor acceptability and overall liking, since they provide a less bitter flavor, besides improving the texture, which contributes to a higher overall liking.

Figure 2 and the equations in Table 1 obtained for each model for acceptability show that well-accepted GFB is possible (acceptability scores ≥ 7) for all attributes when using up to 60% AF, 85% BF, and 82% QF in blends with RF in the composition.

### 3.2. Optimal Gluten-Free Bread Formulations

The results of the desirability function showed that the formulation prepared with 100% RF corresponds to the optimal GFB formulation, with the highest scores for acceptability (Figure S3, in the Supplementary Material).

PCA was used to determine the relations between the physical properties (specific volume and crumb firmness) and the degree of liking of the GFB, presenting the formulations according to similarity. This enabled the finding of the most accepted formulations that contain the highest amount of PF in the composition and present similar characteristics to the optimal formulation (OF) containing 100% RF and to the control formulation (CF) prepared with 50% RF + 50% PS. The results are presented in the supplementary material (Figure S4). The two principal components explain 84.29–93.49% of the experimental variation, indicating strong correlation between the variables investigated, showing that, among the formulations with PF, those prepared with 50% AF, BF, or QF in combination with RF presented physical properties and acceptability closest to OF and CF.

#### 3.2.1. Characterization of Selected Samples

##### Dough Thermomechanical Properties

**Figure 3:**
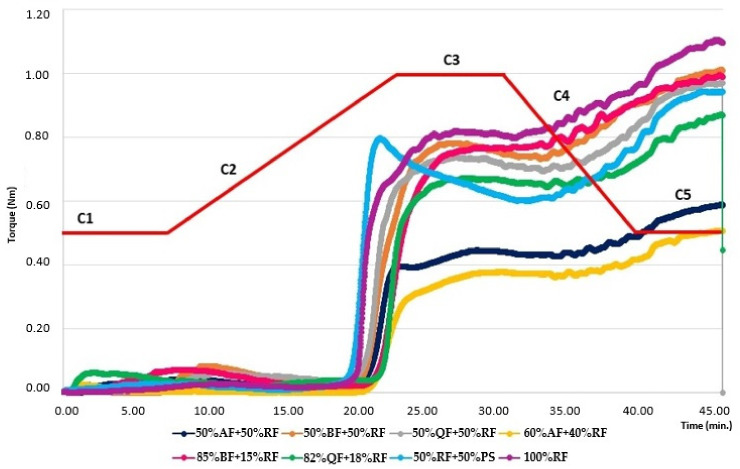
Dough curves of selected gluten-free bread formulations and parameters obtained by Mixolab^®^. Dough mixtures according to the levels of rice flour (RF), potato starch (PS) and pseudocereal flours: amaranth (AF), buckwheat (BF) and quinoa (QF).

Figure 3 shows the dough curves and parameters from Mixolab^®^ for the selected GFB formulations compared to the OF and CF.

No dough shows a significant torque for C1 and C2, which is expected for doughs prepared with GF ingredients. Without gluten, these doughs have a low consistency in the initial stage and no significant protein weakening [29].

GFB doughs containing 100% RF or those prepared with BF showed higher values of C3, C4, and C5 than the CF and doughs containing AF or QF. The doughs with 60% AF + 40% RF and 50% AF + 50% RF presented the lowest C3, C4 and C5 torques.

Alvarez-Jubete et al. (2009) [10] analyzed the peak viscosity of PF compared to RF and found that the amylose content and the particle size are the main causes of these differences. Amaranth presents the lowest content of amylose (<8%), which explains the low gelatinization of the dough containing this PF, while the doughs with higher quantities of RF and BF presented a better gelatinization due to the higher amylose content of these flours [9].

Regarding the C5 parameter, the OF prepared with 100% RF presented the higher values. Santos et al. (2020) [18] evidenced the relation between C5 values with the storage time of the GFB, so based on the results here, doughs with QF or BF show values of C5 near to the CF, except for the dough containing 50% QF + 50% RF, which presented a lower value. As observed in Figure 3, the dough parameters are dependent on the characteristics of the starch sources, which may influence the quality parameters of the final product.

##### Physical Properties and Acceptability Evaluation

**Figure 4:**
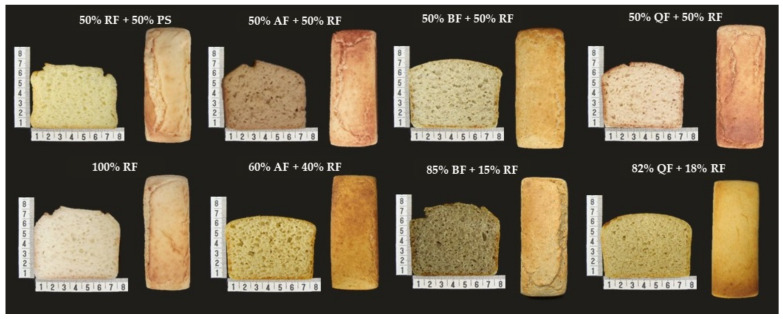
Photographs of central slices and crust of the selected gluten-free bread formulations according to the levels of AF—amaranth flour; BF—buckwheat flour; QF—quinoa flour; RF—rice flour; PS—potato starch.

The selected samples had the appearance, the physical properties and the acceptability evaluated and compared with the 100% RF and 50% RF + 50% PS formulations, as shown in Figure 4 and Table 2.

Overall, the results in Table 2 are consistent with those expected, indicating the good quality of the fitted models.

The group of consumers of the acceptability evaluation were composed of 70% female and 30% male, presenting an average age of 27 ± 10.9 years.

Despite the difference in color between the formulations (Figure 4), high acceptance scores were obtained for this attribute (Table 2), which can indicate a higher custom and acceptance of the consumers to wholemeal products.

The formulations containing BF, QF, and 60% AF showed higher values for specific volume than the OF and CF. However, it should be noted that the crumb firmness obtained in the formulations with BF and QF are higher than those of the OF and CF. Despite the differences in physical properties between these formulations, it was possible to obtain bread with acceptability scores comparable to those of the OF and CF (Table 2).

Table S1, on supplementary material, presents the porosity data of the crumbs of the GFB of the selected formulations. The images of the center of the crumb of the selected formulations are presented in the supplementary material (Figure S5).

The results in Table S1 show that the GFB prepared with 50% QF + 50% RF, 82% QF + 18% RF, and with 85% BF + 15% RF showed the highest values for number of alveoli and the lowest values for mean size, with similar values to the formulation with 100% RF. While the GFB with 50% AF + 50% RF, 60% AF + 40% RF and with 50% BF + 50% RF showed lower number of alveoli and higher values for mean size, being similar to the CF with 50% RF + 50% PS.

Concerning total area, the GFB containing BF showed the highest values; however, they showed no significant difference in relation to the other formulations.

Burešová et al. (2017) [12], comparing the effect of different flours on the characteristics of breads, observed better porosity in breads prepared with BF and QF. While for AF the authors observed low viscosity of dough, relating the difference presented between the PF with the variation of the size of starch granules and gelatinization process of each PF [9]. In the present study, among the PF studied, BF and QF also promoted the greatest positive impact on porosity of breads prepared than AF.

##### Proximate Composition Evaluation

The selected samples had the proximate composition evaluated and compared with the 100% RF and 50% RF + 50% PS formulations, as shown in Table 3.

Based on the composition of the formulations, GFB had higher values of protein, fat and a lower carbohydrate content. Regarding the content of total dietary fiber, the use of PF to develop GFB can contribute to an improvement in the nutritional profile, mainly in the amount of insoluble fiber in the formulations. Alvarez-Jubete, Arendt & Gallagher (2010) [11] reported the nutritional potential of the PF used to develop GFB. The authors noted that the partial replacement of RF with 50% PF resulted in increased content levels of proteins, lipids (high levels of unsaturated fatty acids), dietary fibers and minerals, such as calcium, magnesium, zinc and iron. According to a recent review made by Aguiar et al. (2021a) [5], GFB, available in the market, is mainly classified as low (<3 g/100 g) or source of (>3 g/100 g) fiber content, while the selected GFB formulations can be classified as a product with high fiber content (>6 g/100 g) [30], which evidence the nutritional improvement from the use of whole flours like PF. Therefore, the use of these whole flours in the development of GF products can contribute to a better quality of the diets of CD patients, contributing to a higher consumption of fibers, which can improve the deficient intake of this group and, also, the general population.

### 3.3. Relationships between Dough Properties and Instrumental and Sensory Parameters of GFB

**Figure 5:**
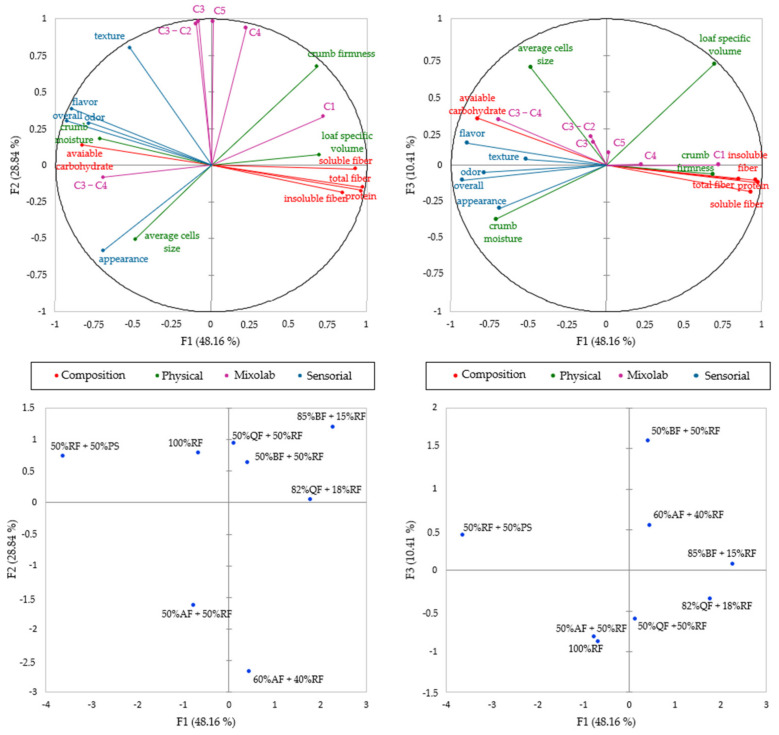
Multiple factor analysis correlating the physical (in green), sensorial (in blue), proximate composition (in red) and Mixolab parameters (in purple) of the selected gluten-free bread formulations. Bread ID: rice flour (RF), potato starch (PS) and pseudocereal flours: amaranth (AF), buckwheat (BF) and quinoa (QF).

Figure 5 shows the relation between variables studied in MFA, having factors sum explained 87.41% of the data total variation.

Figure 5 shows that F1 explained 48.16% of the data variation and positively discriminates all the rheological parameter C1, and the composition parameters protein, insoluble fiber, soluble fiber and the total dietary fiber describing the GFB sample containing 85% BF + 15% RF and 82% QF + 18% RF, induced by the higher amounts of PF in those formulations. Still on F1, the vectors negatively discriminate the variables C3–C4, appearance, odor, flavor, overall, crumb moisture, crumb firmness and available carbohydrate, related to CF (50% RF + 50% PS).

F2 explained 28.84% of the data variation and positively describes the dough parameters C3, C4, C5, C3–C2, and the physical property parameter, texture, related to OF (100% RF) and 50% QF + 50% RF. It negatively describes the same parameters to samples developed with 50% AF + 50% RF and 60% AF + 40% RF, both having higher amounts of AF in the dough.

F3, on the other hand, explained 10.41% of the data variation and was positively discriminated with loaf-specific volume and the average cell size, related to the sample containing 50% BF + 50% RF.

The AFM sorted the selected GFB formulations into four distinct groups (Figure S6, in the Supplementary Material): the first group included samples containing AF (50% AF + 50% RF and 60% AF + 40% RF). The second group was composed of the samples containing the higher amounts of whole PF (85% BF + 15% RF and 82% QF + 18% RF). The third group contained the OF (100% RF) and the formulations with a lower quantity of PF (50% BF + 50% RF and 50% QF + 50% RF). The fourth group was composed of the CF (50% RF + 50% PS).

Based on the data relationship, the combination of PF and RF provides a better dough than those that use PS, resulting in GFB with improved technological, nutritional, and sensory properties, contributing to a better food profile for people who choose or need to follow a GF diet.

## 4. Conclusions

The mixture design showed that the use of PF alone resulted in GFB with low acceptability, due to changes in odor and flavor of the product which the consumers are not so used to.

The results indicate that PF needs to be blended with RF to get possible positive effects, contributing to improved physical properties and better acceptability of the GFB. This shows that adding high PF levels to develop high-quality GFB enriched with protein, fat and dietary fiber is possible.

Blends of 50% AF, BF, or QF with 50% RF (flour basis) to obtain GFB with high acceptance, being similar to GFB formulated with 100% RF and also with the control formulation developed with 50% RF + 50% PS on flour basis.

The mixture design allowed for the determination of the maximum PF proportions that can be used to obtain well-accepted formulations (scores ≥ 7) for appearance, color, odor, texture, and overall liking: 60% AF, 85% BF, and 82% QF in combination with RF.

The promising results of this study indicate an alternative for simultaneous improvement of physical properties, acceptability and nutritional content of GFB, which is very important for the nutrition and health of individuals with restrictions for gluten consumption.

## Figures and Tables

**Table 1:** Predicted model equations for the three mixture designs indicating the effect of each mixture component ^a^ and their interactions on the physical properties and acceptability scores of the gluten-free bread.

Design ^b^	Predicted Model Equations ^c^	R^2^_adj_ (%) ^d^	Model (p) ^e^	Lack of Fit (p) ^e^
	Loaf specific volume cm^3^/g (Y_1_)			
A	Y_1a_ = 1.74RF + 1.43PS + 1.57AF + 0.65RF × PS + 1.42PS × AF	86.79	0.002	0.281
B	Y_1b_ = 1.83RF + 1.59PS + 2.01BF	32.61	0.069	0.017
C	Y_1c_ = 1.72RF + 1.43PS + 1.46QF + 1.11RF × QF + 1.30PS × QF	80.18	0.007	0.063
	Crumb firmness N (Y_2_)			
A	Y_2a_ = 9.88RF + 9.31PS + 4.31AF	73.62	0.001	0.749
B	Y_2b_ = 10.19RF + 11.13PS + 22.69BF	70.82	0.002	0.004
C	Y_2c_ = 8.84RF + 8.97PS + 20.99QF	83.75	0.000	0.277
	Crumb moisture % (Y_3_)			
A	Y_3a_ = 52.28RF + 55.43PS + 53.75AF	98.19	0.000	0.137
B	Y_3b_ = 51.59RF + 55.00PS + 52.11BF	78.63	0.000	0.014
C	Y_3c_ = 52.56RF + 55.63PS + 54.02QF	83.17	0.000	0.461
	Appearance acceptability score (Y_4_)			
A	Y_4a_ = 8.73RF + 7.71PS + 7.25AF + 2.64RF × PS+ 3.46PS × AF	8.55	0.001	0.587
B	Y_4b_ = 9.13RF + 8.13PS + 7.26BF	78.03	0.000	0.255
C	Y_4c_ = 8.75RF + 7.73PS + 7.58QF + 1.83RF × PS + 3.67PS × QF	87.83	0.002	0.269
	Color acceptability score (Y_5_)			
A	Y_5a_ = 8.61RF + 7.89PS + 7.77AF + 2.91RF × AF	66.25	0.033	0.571
B	Y_5b_ = 8.98RF + 8.07PS + 6.97BF	79.72	0.000	0.416
C	Y_5c_ = 8.57RF + 7.91PS + 7.43QF + 3.04RF × QF	73.85	0.016	0.237
	Odor acceptability score (Y_6_)			
A	Y_6a_ = 8.59RF + 8.62PS + 7.62AF	65.42	0.003	0.251
B	Y_6b_ = 8.50RF + 8.34PS + 7.30BF	77.10	0.001	0.607
C	Y_6c_ = 8.41RF + 8.33PS + 6.74QF	84.23	0.000	0.815
	Texture acceptability score (Y_7_)			
A	Y_7a_ = 8.09RF + 7.07PS + 4.87AF + 3.58RF × PS + 3.55RF × AF	92.02	0.001	0.229
B	Y_7b_ = 8.63RF + 7.30PS + 6.99BF	59.59	0.007	0.473
C	Y_7c_ = 8.37RF + 7.78PS + 7.46QF	05.71	0.311	0.043
	Flavor acceptability score (Y_8_)			
A	Y_8a_ = 7.87RF + 7.88PS + 5.62AF + 3.24RF + AF + 3.19PS + AF	91.63	0.001	0.878
B	Y_8b_ = 7.89RF + 7.84PS + 6.51BF + 2.04RF × PS + 2.35RF × BF	93.63	0.000	0.459
C	Y_8c_ = 8.30RF + 8.29PS + 6.72QF	58.00	0.008	0.642
	Overall liking (Y_9_)			
A	Y_9a_ = 8.14RF + 7.80PS + 5.78AF + 3.51RF × AF + 2.78 PS × AF	90.34	0.001	0.440
B	Y_9b_ = 8.20RF + 7.67PS + 7.88BF + 2.45RF × PS − 1.99PS × BF	84.07	0.003	0.230
C	Y_9c_ = 8.40RF + 8.27PS + 6.87QF	61.38	0.005	0.411

^a^ Mixture components: RF = rice flour, PS = potato starch, AF = amaranth flour, BF = buckwheat flour, QF = quinoa flour. ^b^ Design: amaranth (A), buckwheat (B) and quinoa (C). ^c^ Only the coefficients significant at a *p* < 0.05 level were selected for the predicted model construction. ^d^ *R*^2^_adj_ adjusted coefficient of determination. ^e^ Significance of the Model and Lack of fit. *p* = probability level.

**Table 2:** Predicted and measured values to physical properties and sensory analysis of the selected gluten-free bread formulations.

Parameters		Gluten-Free Bread Formulations ^a^							
		50% AF + 50% RF	50% BF + 50% RF	50% QF + 50% RF	60% AF + 40% RF	85% BF + 15% RF	82% QF + 18% RF	100% RF	50% RF + 50% PS
Physical properties									
Specific volume (cm^3^/g) ^b^	PV *	1.80 (1.67–1.94)	SV	1.81 (1.65–1.97)	1.80 (1.65–1.94)	SV	1.75 (1.54–1.96)	1.77 (1.61–1.93)	1.91 (1.64–2.18)
	MV *	1.71 ^e^ (1.67–1.72)	2.10 ^a^ (1.99–2.23)	1.86 ^d^ (1.81–1.91)	1.,94 ^bc^ (1.91–1.99)	2.00 ^b^ (1.97–2.04)	1.88 ^cd^ (1.87–1.89)	1.69 ^e^ (1.58–1.79)	1.72 ^e^ (0.13–1.67)
Crumb firmness (N) ^c^	PV *	7.09 (6.17–8.02)	SV	14.91 (13.34–16.49)	6.54 (5. 58–7.50)	SV	18.80 (16.75–20.85)	8.90 (7.33–10.48)	8.84 (6.24–11.43)
	MV *	7.69 ^e^ (7.13–8.24)	12.04 ^c^ (11.36–12.71)	14.73 ^b^ (13.96–15.50)	7.24 ^e^ (6.85–7.61)	16.44 ^a^ (15.83–17.05)	14.03 ^b^ (13.00–5.07)	10.63 ^d^ (9.84–11.42)	13.48 ^b^ (12.73–14.23)
Sensory acceptance ^d^									
Appearance	PV *	SV	8.20 (7.94–8.45)	8.53 (8.17–8.89)	SV	7.54 (7.20–7.88)	8.22 (7.75–8.69)	8.63 (8.38–8.88)	9.13 (8.71–9.54)
	MV *	8.76 ^a^ (8.42–9.09)	8.39 ^ab^ (7.96–8.82)	8.82 ^a^ (8.49–9.15)	8.87 ^a^ (8.53–9.21)	7.83 ^b^ (7.28–8.38)	8.14 ^ab^ (7.66–8.62)	8.74 ^a^ (8.29–9.19)	8.75 ^a^ (8.34–9.16)
Color	PV *	SV	7.97 (7.71–8.23)	8.41 (8.06–8.77)	SV	7.27 (6.92–7.62)	8.10 (7.64–8.57)	8.52 (8.26–8.78)	8.98 (8.55–9.40)
	MV *	8.80 ^a^ (8.03- 8.98)	7.88 ^bc^ (7.38–8.38)	8.83 ^a^ (8.49–9.18)	8.96 ^a^ (8.69–9.24)	7.43 ^c^ (6.91–7.95)	8.16 ^abc^ (7.72–8.60)	8.50 ^ab^ (8.03–8.78)	8.59 ^ab^ (8.12–9.06)
Odor	PV *	SV	7.90 (7.71–8.08)	7.58 (7.37–7.79)	SV	7.48 (7.23–7.73)	7.04 (6.77–7.31)	8.45 (8.26–8.63)	8.50 (8.19–8.80)
	MV *	7.94 ^a^ (7.40–8.50)	7.62 ^a^ (7.02–8.21)	8.11 ^a^ (7.60–8.62)	7.70 ^a^ (7.10–8.31)	7.60 ^a^ (7,05–8,14)	7.90 ^a^ (7.37–8.42)	8.41 ^a^ (7.92–8.89)	8.44 ^a^ (7.97–8.91)
Texture	PV *	7.19 (6.65–7.72)	SV	SV	7.01 (6.30–7.41)	SV	SV	7.96 (7.61–8.32)	8.63 (8.05–9.21)
	MV *	7.67 ^a^ (7.97–8.85)	8.06 ^a^ (7.50–8.61)	7. 72 ^a^ (7.19–8.25)	7.36 ^a^ (6.84–7.84)	7.96 ^a^ (7.48–8.44)	7.44 ^a^ (6.95–7.93)	8.41 ^a^ (7.96–8.85)	8.18 ^a^ (7.71–8.64)
Flavor	PV *	7.36 (6.91–7.81)	7.59 (7.31–7.87)	SV	7.14 (6.68–7.61)	7.06 (6.68–7.44)	SV	8.23 (7.96–8.52)	8.35 (7.89–8.82)
	MV *	7.69 ^a^ (6.67–7.99)	8.15 ^a^ (7.52–8.78)	8.23 ^a^ (7.81–8.65)	7.33 ^a^ (6.78–7.91)	7.35 ^a^ (7.15–8.23)	7.32 ^a^ (6.74–7.89)	8.44 ^a^ (8.02–8.86)	7.83 ^a^ (7.32–8.34)
Overall liling	PV *	7.54 (7.13–8.04)	7.98 (7.71–8.24)	SV	7.36 (6.89–7.82)	7.69 (7.33–8.05)	SV	8.05 (7.79–8.20)	8.39 (7.94–8.83)
	MV *	8.00 ^a^ (7.55–8.44)	8.13 ^a^ (7.70–8.55)	8.33 ^a^ (7.99–8.67)	7.56 ^a^ (7.00–8.13)	7.58 ^a^ (7.11–8.06)	7.61 ^a^ (7.17–8.05)	8.43 ^a^ (8.04–8.82)	8.11 ^a^ (7.68–8.55)

^a^ Bread IDs: AF—amaranth flour; BF—buckwheat flour; QF—quinoa flour; RF—rice flour; PS—potato starch. The numbers indicate the ingredient proportions in the flour weight basis (g/100 g). Values are means ± standard deviations ^b^ (*n* = 3), ^c^ (*n* = 6), ^d^ (*n* = 54). * PV: predicted values, MV: measured values. Values followed by different letters in each line are significantly different (*p* < 0.05).

**Table 3:** Proximate composition of the selected gluten-free bread (GFB) formulations, compared with white GFB developed with rice flour (RF) and potato starch (PS) (g/100 g of food as eaten).

	Gluten-Free Bread Formulations ^a^							
	50% AF + 50% RF	50% BF + 50% RF	50% QF + 50% RF	60% AF + 40% RF	85% BF + 15% RF	82% QF + 18% RF	100% RF	50% RF + 50% PS
Moisture	47.03 + 0.02 ^bcd^	47.05 + 0.13 ^bcd^	46.22 + 0.05 ^cd^	47.47 + 0.27 ^abc^	47.41 + 0.06 ^abcd^	47.58 + 0.03 ^ab^	46.16 + 0.26 ^d^	48.59 + 1.24 ^a^
Ash	1.71 + 0.03 ^c^	1.66 + 0.00 ^d^	1.80 + 0.00 ^b^	1.80 + 0.02 ^b^	1.00 + 0.01 ^g^	2.03 + 0.01 ^a^	1.40 + 0.00 ^e^	1.32 + 0.00 ^f^
Protein	6.90 + 0.03 ^d^	6.72 + 0.05 ^e^	7.67 + 0.02 ^b^	6.92 + 0.07 ^d^	7.29 + 0.06 ^c^	8.02 + 0.08 ^a^	5.12 + 0.00 ^f^	4.10 + 0.09 ^g^
Fat	5.13 + 0.47 ^ab^	4.78 + 0.38 ^abc^	4.05 + 0.07 ^de^	4.71 + 0.14 ^bcd^	4.17 + 0.17 ^cd^	3.42 + 0.13 ^e^	5.39 + 0.11 ^a^	4.17 + 0.03 ^cd^
Total dietary fiber	10.39 + 0.47 ^c^	12.74 + 0.51 ^b^	10.78 + 0.20 ^c^	11.41 + 0.07 ^c^	13.40 + 0.47 ^ab^	14.37 + 0.57 ^a^	7.66 + 0.18 ^d^	4.89 + 0.12 ^e^
Insoluble fiber	7.82 + 0.17 ^d^	10.24 + 0.33 ^b^	8.05 + 0.14 ^cd^	8.61 + 0.29 ^c^	10.31 + 0.12 ^b^	11.47 + 0.36 ^a^	5.50 + 0.22 ^e^	3.10 + 0.10 ^f^
Soluble fiber	2.56 + 0.48 ^abc^	2.50 + 0.19 ^abc^	2.74 + 0.34 ^abc^	2.80 + 0.26 ^ab^	3.09 + 0.37 ^a^	2.91 + 0.22 ^ab^	2.15 + 0.04 ^bc^	1.78 + 0.13 c
Available carbohydrate	28.83 + 0.84 ^cd^	27.05 + 0.59 ^de^	29.46 + 0.10 ^c^	27.69 + 0.16 ^cde^	26.74 + 0.66 ^e^	24.57 + 0.55 ^f^	34.27 + 0.43 ^b^	36.93 + 1.18 ^a^

^a^ Bread IDs: AF—amaranth flour; BF—buckwheat flour; QF—quinoa flour; RF—rice flour; PS—potato starch. The numbers indicate the ingredient proportions in the flour weight basis (g/100 g). Values are means ± standard deviations (*n* = 3). Values followed by different letters in each line are significantly different (*p* < 0.05).

## Data Availability

Not applicable.

## Supplementary Materials

The following supporting information can be downloaded at: www.mdpi.com/article/10.3390/foods11060848/s1, Figure S1: Flow chart of the experimental design. * Formulation ID: rice flour (RF), potato starch (PS) and pseudocereals flours—amaranth flour (AF), buckwheat flour (BF) or quinoa flour (QF). Figure S2: Scanned images of the gluten-free bread (GFB) formulations obtained from the experimental mixture designs. * Bread ID: rice flour (RF), potato starch (PS) and pseudocereals flours—amaranth flour (AF), buckwheat flour (BF) or quinoa flour (QF). Figure S3: Profiles for predicted mixture experimental design of rice flour (RF), potato starch (PS) and pseudocereal flour (amaranth (AF), buckwheat (BF) or quinoa (QF)) and the desirability level for acceptability factor for optimum gluten-free bread. Figure S4: Principal component analysis of mixture design to evaluate the effect of rice flour—RF, potato starch—PS and pseudocereal flour (amaranth flour—AF (A), or buckwheat flour—BF (B) or quinoa flour—QF (C)) on physical properties and acceptability of gluten-free breads. Figure S5: Crumb porosity of gluten-free bread formulations selected from the mixture designs. Figure S6. Dendrogram obtained by hierarchical cluster analysis for data of selected gluten-free bread formulations. Table S1: Crumb porosity of gluten-free bread formulations selected from a mixture design to study the effects of pseudocereal flour: amaranth (AF), buckwheat (BF) and quinoa (QF) based gluten-free breads, comparing with white formulations developed with rice flour (RF) and potato starch (PS).
