# Supplementary material for: Defining Amaranth, Buckwheat and Quinoa Flour Levels in Gluten-Free Bread: A Simultaneous Improvement on Physical Properties, Acceptability and Nutrient Composition through Mixture Design
Source: Foods. 2022 Mar 16;11(6):848. doi: 10.3390/foods11060848 (PMC8954203; doi:10.3390/foods11060848)
Supplement: Supplementary file 1 [file foods-11-00848-s001.zip › foods-1615162-supplementary.pdf]

Trials	Component proportion in flour/ starch blend[1]			Component levels in bread formulation (g/ 100g of dough)		
	x_1	x_2	x_3	RF	PS	PF
1	1.00	0.00	0.00	35.8	0.00	0.00
2	0.00	1.00	0.00	0.00	35.8	0.00
3	0.00	0.00	1.00	0.00	0.00	35.8
4	0.50	0.50	0.00	17.9	17.9	0.00
5	0.50	0.00	0.50	17.9	0.00	17.9
6	0.00	0.50	0.50	0.00	17.9	17.9
7	0.33	0.33	0.33	11.9	11.9	11.9
8	0.33	0.33	0.33	11.9	11.9	11.9
9	0.33	0.33	0.33	11.9	11.9	11.9
10	0.66	0.17	0.17	23.8	6.0	6.0
11	0.17	0.66	0.17	6.0	23.8	6.0
12	0.17	0.17	0.66	6.0	6.0	23.8

[1] x_1= Rice flour (RF), x_2= Potato starch (PS), x_3= Pseudocereal flour (PF) - amaranth (AF), buckwheat (BF) and quinoa (QF).

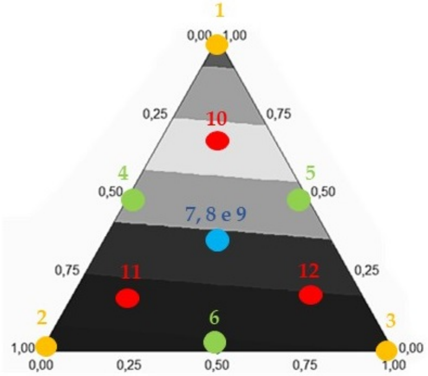

Figure S1. Simplex centroid design used to optimize pseudocereal based gluten-free bread formulations. Design table and ternary plot: Trials 1-3 constituted of single components (100%), trails 4-6 to the binary blends prepared with 50% of each component, trials 7-9 to ternary blend consisting of the combination of 33.3% of each component, representing the central point of the model, which was made in three repetitions, and trials 10-12 to the ternary blend consisting of the combination of 66% of each component and 17% of the others, corresponding to the axial points.

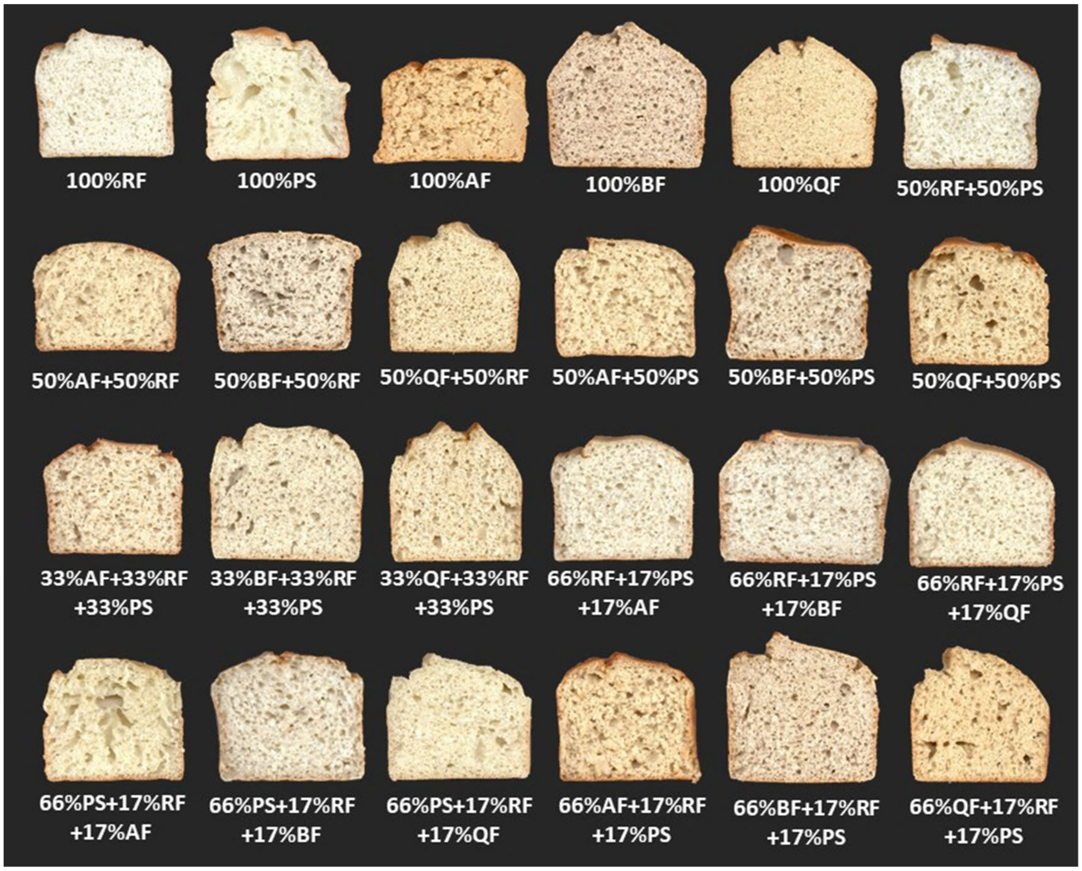

Figure S2. Scanned images of the gluten-free bread (GFB) formulations obtained from the experimental mixture designs. * Formulation ID: AF- amaranth flour; BF- buckwheat flour; QF- quinoa flour; RF- rice flour; PS- potato starch. The numbers indicate the ingredient proportions in the flour weight basis (g/ 100 g).

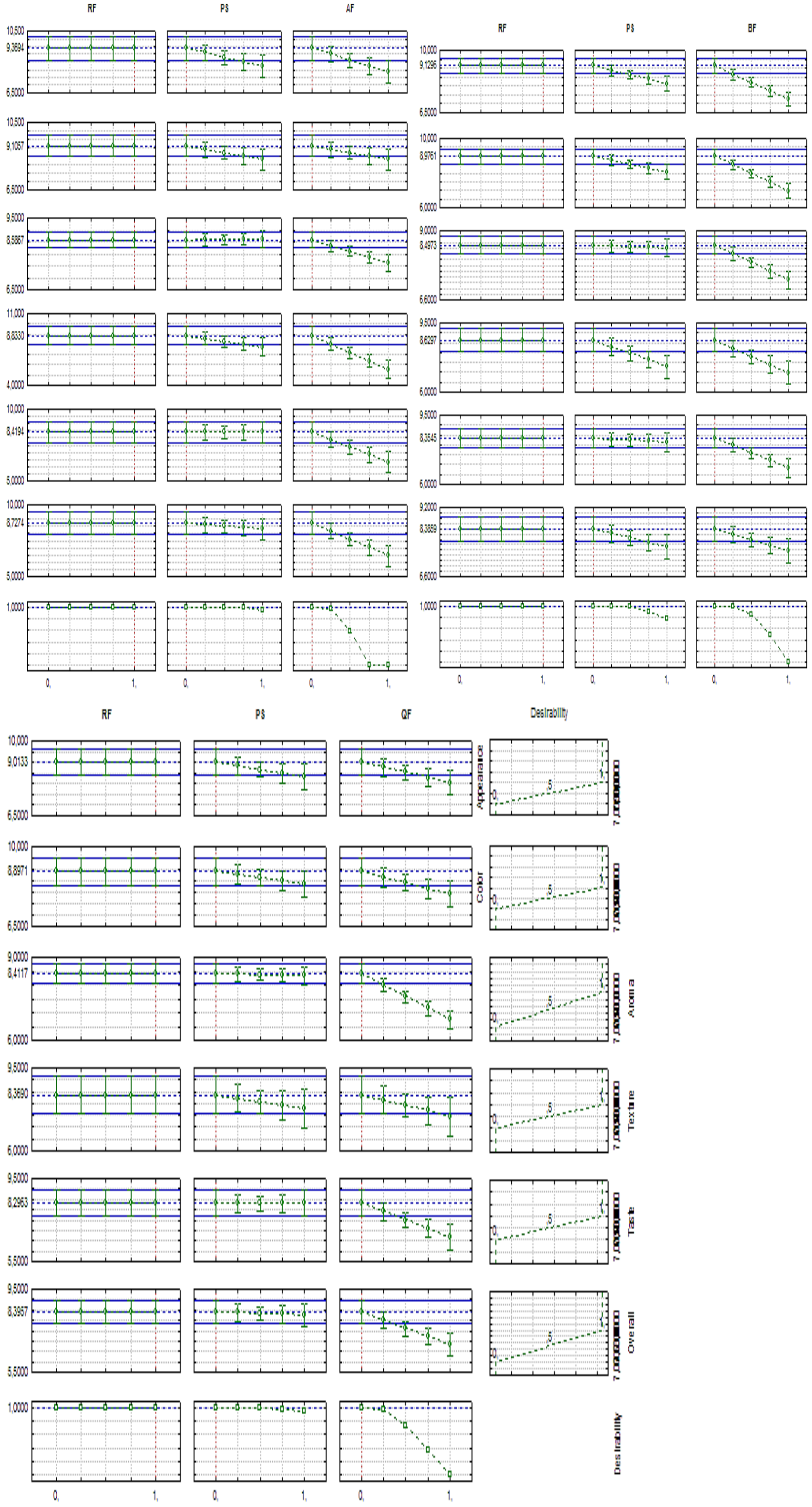

Figure S3. Profiles for predicted mixture experimental design of rice flour (RF), potato starch (PS) and pseudocereal flour (amaranth (AF), buckwheat (BF) or quinoa (QF)) and the desirability level for acceptability factors for optimum gluten-free bread.

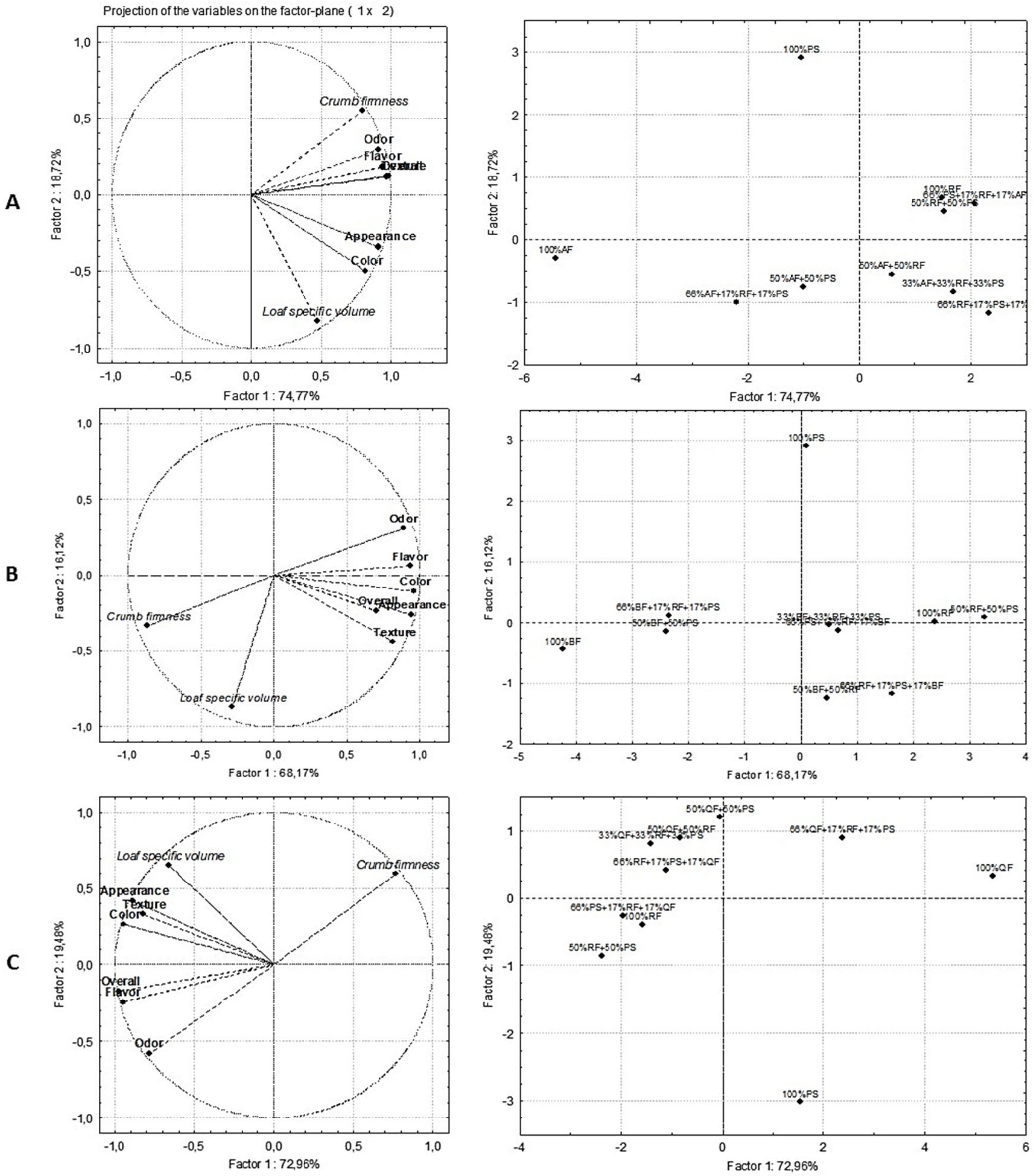

Figure S4. Principal Component Analysis of mixture design to evaluate the effect of rice flour - RF, potato starch - PS and amaranth flour – AF (A), or buckwheat flour – BF (B) or quinoa flour – QF (C) on physical properties and acceptability of gluten-free breads.

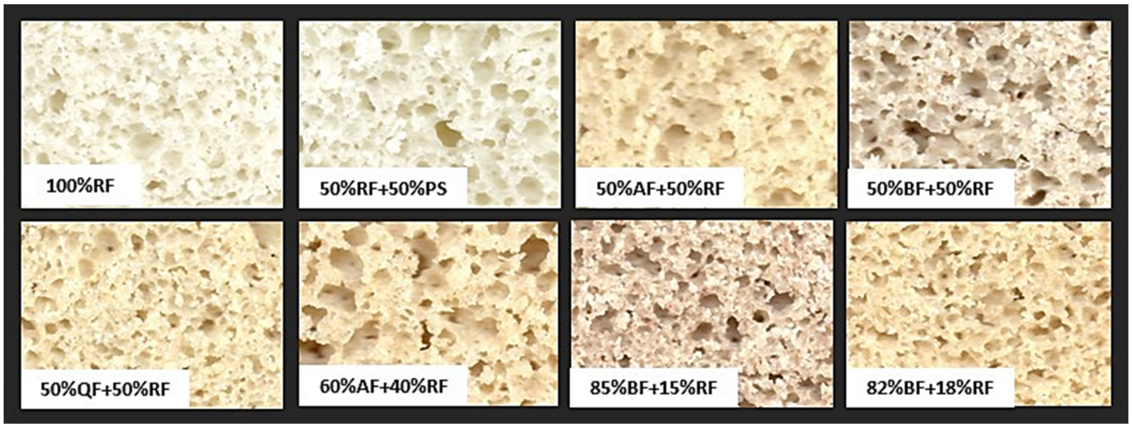

Figure S5. Crumb porosity of gluten-free bread formulations selected from the mixture designs. *Bread ID: rice flour (RF), potato starch (PS) and pseudocereal flours - amaranth flour (AF), buckwheat flour (BF) or quinoa flour (QF). The numbers indicate the ingredient proportions in the flour weight basis (g/ 100g).

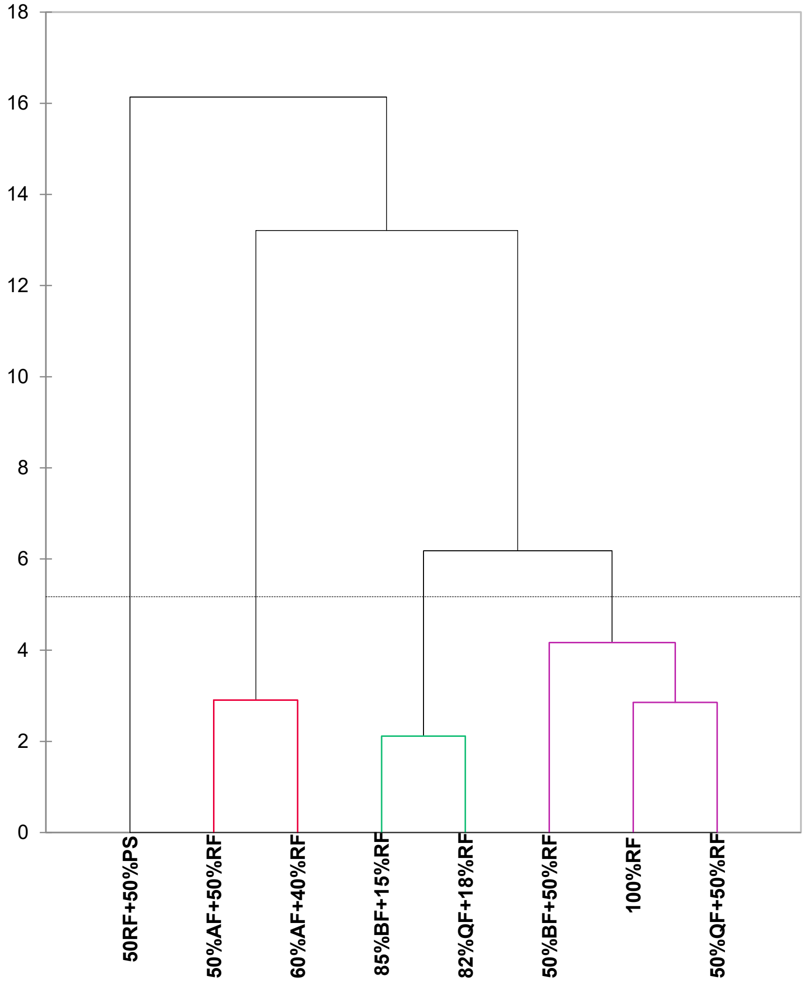

Figure S6. Dendrogram obtained by hierarchical cluster analysis for data of seletecd gluten-free bread formulations. * Formulation ID: AF- amaranth flour; BF- buckwheat flour; QF- quinoa flour; RF- rice flour; PS- potato starch. The numbers indicate the ingredient proportions in the flour weight basis (g/ 100g).

Table S1. Crumb porosity of gluten-free bread formulations selected from a mixture design to study the effects of pseudocereal flour: amaranth (AF), buckwheat (BF) and quinoa (QF) based gluten-free breads, comparing with white formulations developed with rice flour (RF) and potato starch (PS).

Parameter	50%AF+ 50%RF*	50%BF+ 50%RF	50%QF+ 50%RF	60%AF+ 40%RF	85%BF+ 15%RF	82%QF+ 18%RF	100%RF	50%RF+ 50%PS
Number of alveoli/cm^2	146.50^{b} ± 0.71	135.50^{b} ± 9.19	244.50^{a} ± 3.54	125.00^{b} ± 2.83	224.00^{a} ± 15.56	223.00^{a} ± 8.49	134.50^{b} ± 3.54	235.50^{a} ± 3.54
Average size of alveoli (mm^2)	0.0152^{bc} ± 0.0002	0.0197^{a} ± 0.0017	0.0088^{d} ± 0.0006	0.0185^{ab} ± 0.0008	0.0112^{cd} ± 0.0009	0.0095^{d} ± 0.0015	0.0185^{ab} ± 0.0001	0.0096^{d} ± 0.0013
Total area alveoli (%)	25.34^{a} ± 0.48	30.49^{a} ± 0.68	24.56^{a} ± 1.97	26.40^{a} ± 0.58	28.76^{a} ± 0.40	24.21^{a} ± 2.91	28.38^{a} ± 0.98	25.76^{a} ± 3.12

Values are the means and 95% confidence intervals.

Values followed by different superscripts in each row are significantly different ($p<.05$).

* The numbers indicate the ingredient proportions in the flour weight basis (g/ 100g).
